# Use of Negative Pressure Wound Therapy With Automated, Volumetric Instillation for the Treatment of Extremity and Trunk Wounds: Clinical Outcomes and Potential Cost-Effectiveness

**Published:** 2014-11-03

**Authors:** Allen Gabriel, Kevin Kahn, Riyad Karmy-Jones

**Affiliations:** ^a^PeaceHealth Medical Group Plastic Surgery, Vancouver, Washington; ^b^Rebound, Vancouver, Washington; ^c^PeaceHealth Medical Group Thoracic and Vascular Surgery, Vancouver, Washington

**Keywords:** cost-effectiveness, dwell time, health economics, instillation therapy, negative pressure wound therapy with instillation

## Abstract

**Objective:** A growing body of literature supports use of negative pressure wound therapy (NPWT) with instillation and dwell time (NPWTi-d) with positive clinical outcomes and potential cost savings. A retrospective analysis was performed to compare clinical outcomes of wounds treated with NPWTi-d versus NPWT and to estimate cost-differences between treatments based on clinical outcomes. **Methods:** Data were extracted from records of patients with extremity or trunk wounds treated with NPWT (n = 34) or NPWTi-d using saline or polyhexanide (n = 48). On the basis of outcomes data, a hypothetical economic model using cost assumptions was created to calculate cost savings for NPWTi-d (related to) number of debridements and length of therapy. Operating room debridement cost was $3393 according to Granick et al. Daily therapy cost for each modality was $194.80 (NPWTi-d) and $106.08 (NPWT) based on internal company information. **Results:** Results showed significant differences (*P* < 0.0001) between NPWTi-d and NPWT patients, respectively, for the following: mean operating room debridements (2.0 vs 4.4), mean hospital stay (8.1 vs 27.4 days), mean length of therapy (4.1 vs 20.9 days), and mean time to wound closure (4.1 vs 20.9 days). Hypothetical economic model showed potential average reduction of $8143 for operating room debridements between NPWTi-d ($6786) and NPWT ($14,929) patients. There was a $1418 difference in average therapy costs between groups ($799/NPWTi-d vs $2217/NPWT). **Conclusions:** In this study, NPWTi-d appeared to assist in wound cleansing and exudate removal, which may have allowed for earlier wound closure compared to NPWT. Hypothetical economic model findings illustrate potential cost-effectiveness of NPWTi-d compared to NPWT.

Direct and indirect costs of wound care contribute to overall health care expenditures in the United States^1^ and may increase when complications, such as infection or edema, cause healing delays. Balancing the benefits of lower cost versus higher cost wound healing technologies is a challenging, yet critical, component of determining cost-effectiveness. While negative pressure wound therapy (NPWT) is often associated with higher-cost advanced wound care therapies, overall savings in direct and indirect cost have been reported with NPWT due to resulting decreases in operating room (OR) visits, earlier wound closure, reduced hospital stay, fewer required dressing changes, and improved limb salvage rates.^2^^-^8

While reduced rates of infection have been reported with use of adjunctive NPWT in acute and chronic infected and critically colonized wounds, results have been somewhat mixed. NPWT has been reported to reduce nonfermentative gram-negative bacilli, yet wounds culturing *Staphylococcus aureus* have shown increased bacterial levels over time with NPWT.^9^ Additional limitations of NPWT have included inability to clear thick exudates through the foam, as well as painful dressing removal. A next-generation NPWT system is now available that facilitates automated, volumetric control of instilled topical wound solutions in combination with NPWT. This NPWT system with instillation and a dwell time (NPWTi-d) differs from other irrigation and instillation systems in that a timed, predetermined volume of topical solution is intermittently delivered, versus continuously fed, and allowed to dwell in the wound bed while NPWT is paused, for a user-selected period of time before NPWT is resumed.^10^ Although NPWT systems with intermittent instillation or continuous irrigation have been commercially available since 2004, they have historically been considered cumbersome and time-consuming to use. The new volumetrically controlled NPWTi-d system is fully automated with consistent, controlled fluid instillation.

In addition to providing the labeled therapeutic benefits of the delivered solution, the addition of an instilled solution assists with wound cleansing and lowers wound fluid viscosity, which in turn facilitates more efficient removal of exudates and infectious material utilizing NPWT.^11^^-^13 Positive results have been reported with the combined use of adjunctive NPWT and instillation of topical solutions in wounds at risk for compromised healing, such as noninfected open fractures, infections of bone and/or soft tissue, and chronic ulcers, which made lead to a potential cost benefit.^11^^,^^14^^-^18

Therefore, we conducted a retrospective data analysis to compare the outcomes of patients with extremity and trunk wounds treated with standard NPWT versus NPWTi-d with volumetric fluid instillation and to estimate differences in costs for the 2 treatment arms based on the outcomes. Variables included in the comparative analysis were number of surgical debridements, hospital stay, length of therapy (LOT) and time to wound closure.

## METHODS

Approval for this study was granted by the System Internal Review Board of PeaceHealth Southwest Medical Center in Vancouver, Washington. Medical records of patients treated between January 1, 2010, and May 31, 2013, at PeaceHealth Southwest Medical Center (Vancouver, Washington) were retrospectively reviewed. Patients from age 21 to 80 with an infected or critically colonized wound treated with either NPWTi-d (V.A.C. VeraFlo Therapy, KCI, San Antonio, Texas) or standard NPWT (V.A.C. Therapy, KCI, San Antonio, Texas) were included in the analysis. Patients with pressure ulcers or wounds with infected hardware or implants were excluded from the analysis.

All patients were treated with a similar protocol by one investigator. Wounds were debrided, and systemic antibiotics were administered prior to any therapy. For patients receiving NPWTi-d, a reticulated open-cell foam (ROCF) dressing specifically designed for use with NPWTi-d (ROCF-V; V.A.C. VeraFlo Dressing, KCI, San Antonio, Texas) was placed over the wound. Saline or polyhexanide (Prontosan, B. Braun Medical Inc, Bethlehem, Pennsylvania) was instilled to fill the foam with a set soak time ranging from 1 to 60 seconds, followed by NPWT of −125 mm Hg for 1 to 2 hours. For patients receiving traditional NPWT, black or silver foam (V.A.C. GranuFoam Dressing, V.A.C. GranuFoam Silver Dressing, KCI, San Antonio, Texas) was placed in the wound with −125 mm Hg continuous pressure applied. Dressing changes occurred every 2 to 3 days for both groups.

A hypothetical economic model was then developed to estimate the average overall costs of NPWTi-d versus NPWT during the study timeframe. The mean number of surgical debridements and mean LOT endpoints from the NPWT and NPWTi-d group analysis were used in the model. Cost of a surgical OR debridement was estimated to be $3393, based on a published report by Granick and colleagues.^19^ Product costs were calculated from the manufacturer data, and the actual dressing/canister change frequency reported during the study timeframe for each study arm was used to calculate costs in the economic model.

The daily cost of NPWTi-d assumed an average daily cost of therapy unit ($63.25), and use of 0.8 canisters per day ($42.49/canister), 3 medium instillation dressings ($115/dressing) over average hospital stay (4.1 days), and 1 cassette ($55/cassette) over average hospital stay (4.1 days). Instillation solution costs were excluded from this model. Daily cost of NPWTi-d was calculated as follows:


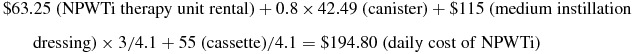


The daily cost of standard NPWT assumed an average daily cost of therapy unit ($63.25) and usage of 0.33 canisters per day ($42.49/canister). Dressing usage was based on 78.57% versus 21.43% mix of medium silver dressings ($71.80/dressing) and medium black dressings ($50.40/dressing), respectively, changed 3 times per week. Daily cost of NPWT was calculated as follows:


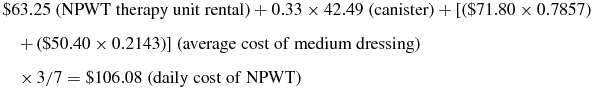


Total therapy cost for each of the 2 treatment arms was computed as


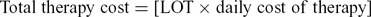


Categorical data were compared using Fisher exact test (2-tailed) and continuous variables were compared using Wilcoxon rank sum test (2-sided). All analyses were performed using Statistical Analysis System (SAS Software; SAS Institute Inc, Cary, North Carolina) version 9.3.

## RESULTS

Forty-eight patients received NPWTi-d and were compared to a historical control group of 34 patients who received standard NPWT. Patient demographic variables were similar between the 2 groups (Table 1). Comorbidities for both groups included obesity and diabetes.

Clinical results showed patients who received NPWTi-d required fewer surgical OR debridements (2.0 vs 4.4) and experienced a shorter average length of hospital stay (8.1 vs 27.4 days), LOT (4.1 vs 20.9 days), and time to wound closure (4.1 vs 20.9 days) (*P* < 0.0001), compared to patients treated with NPWT (Table 2).

The hypothetical model showed an average reduction of $8143 for OR debridement costs with NPWTi-d versus NPWT patients ($6786 vs $14,929, respectively), based on average actual frequency of OR debridements (2.0 vs 4.4) received by the NPWTi-d versus NPWT groups (Table 3). When average LOT was multiplied by the daily cost of NPWTi-d therapy, average therapy cost was $1418 lower for the NPWTi-d group ($799 for NPWTi-d vs $2,217 for NPWT) (Table 3).

### Case Studies

#### Case Study 1

**Figure 1:**
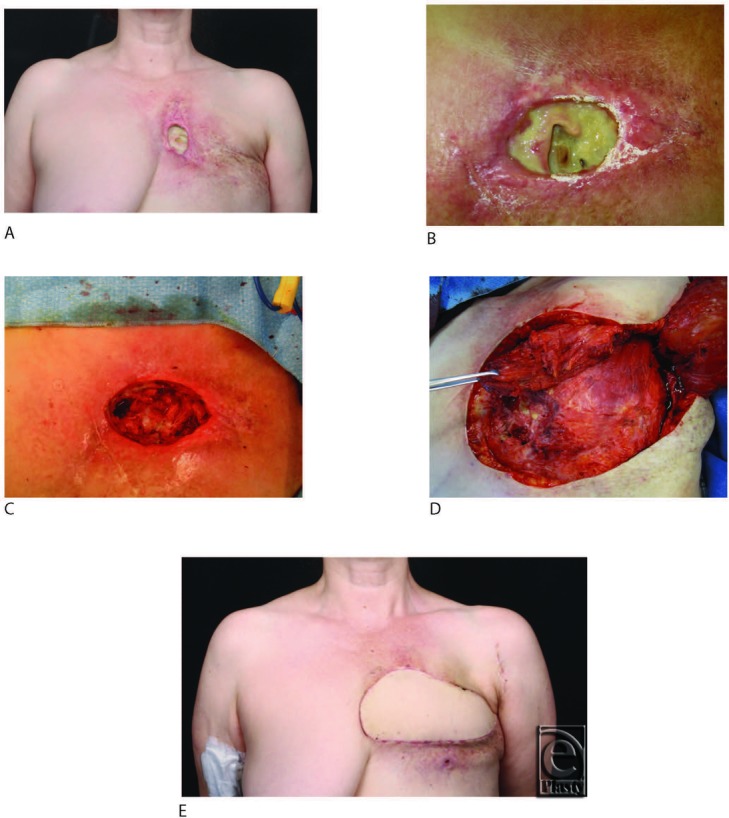
(*a*) Radiated chest wound. (*b*) Chest wound at initial presentation. (c) Wound after debridement of rib and cartilage and 3 days of NPWTi-d. (*d*) Excision of radiated skin at end of NPWTi-d. (*e*) 6 weeks following chest wall reconstruction with latissimus flap. NPWTi-d indicates negative pressure wound therapy with instillation and dwell time.

A 43-year-old woman presented with an infected chest wound after radiation (Figs 1A and 1B). NPWTi-d was initiated with polyhexanide instilled until the foam was filled, followed by a soak time of 30 seconds. Instillation was repeated every hour followed by continuous negative pressure at −125 mm Hg for 3 days (Fig 1C). After NPWTi-d was terminated, the radiated skin was excised (Fig 1D) and a latissimus flap was performed (Fig 1E).

#### Case Study 2

**Figure 2:**
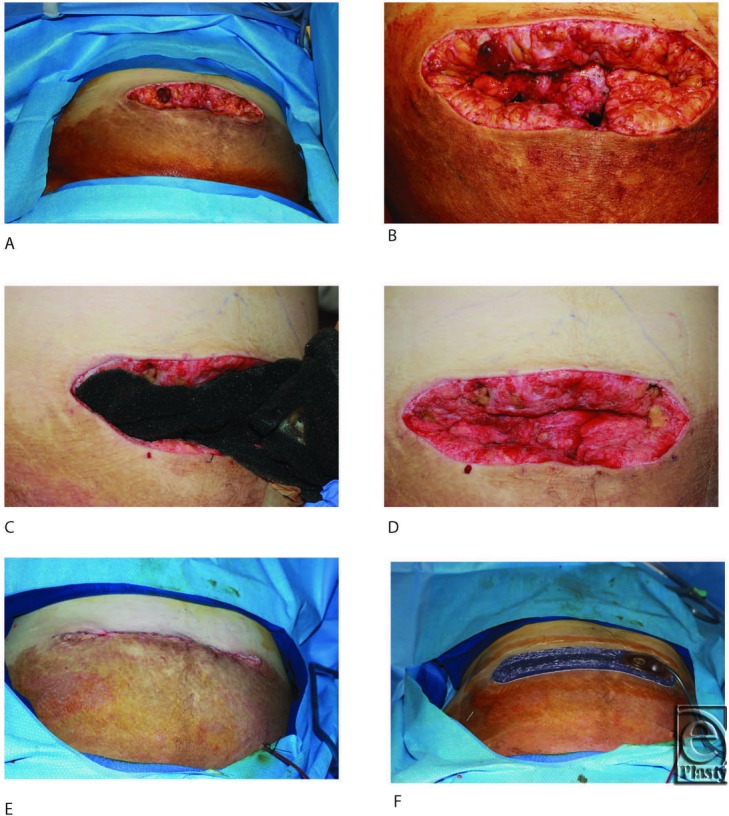
(*a*) Postoperative day 1 following debridement. (*b*) Postoperative day 1 following debridement (close up). (*c*) Application of ROCF-V. (*d*) Postoperative day 4 with 3 days of NPWTi-d. (*e*) Primary closure of wound. (*f*) Application of incisional NPWT over closed incision. NPWT indicates negative pressure wound therapy; NPWTi-d, negative pressure wound therapy with instillation and dwell time.

A 43-year-old obese woman presented with an infected, dehisced abdominal wound. Systemic antibiotics were administered, and the wound was debrided of nonviable tissue (Fig 2A and 2B). NPWTi-d was initiated with a ROCF-V dressing (Fig 2C) and intermittent instillation of saline every 2 hours for 5 days, during which time 2 debridements/dressing changes were performed (Fig D). The wound was surgically closed on day 5 without further complications (Fig 2E and 2F).

## DISCUSSION

In this retrospective comparative analysis, automated instillation of solutions with NPWT resulted in fewer required OR debridements, faster time to closure, and shorter hospital stay and LOT, compared to standard NPWT treatment. Wounds treated with NPWTi-d appeared more beefy red, as if they had taken a bath. Dressing changes were notably easier and less painful with NPWTi-d versus NPWT, and odor was substantially reduced with solution instillation, including saline. Based on a hypothetical economic model introduced in this article, the endpoint differences translated into reduced surgical debridement costs and overall therapy costs with use of NPWTi-d, despite its increased material costs. Although a handful of other retrospective studies have compared outcomes of NPWT- versus NPWTi-d-treated wounds, this is the first analysis that attempts to estimate potential hospital and health care system costs associated with the use of NPWTi-d.

Although our outcomes display wider differences between the 2 groups, our overall results agree with a recent retrospective analysis conducted by Kim et al,^20^ whereby NPWTi-d with intermittently instilled polyhexanide was compared to standard NPWT for the adjunctive treatment of 142 acutely and chronically infected wounds. The number of operative visits was significantly lower (*P* ≤ 0.05) for patients treated with instillation and NPWT with 6- and 20-minute soak times compared with patients who received NPWT and no instillation. Hospital stay was also significantly shorter (*P* ≤ 0.05) for the 20-minute dwell time group compared with only NPWT-treated.

Authors of smaller studies have also reported positive findings with NPWT and instillation versus standard NPWT treatment of wounds. Brinkert et al^21^ used the NPWTi-d system in a case series of 131 acute and chronic wounds treated with NPWT and saline for a mean period of 12 to 19 days. A total of 128 of 131 wounds could be closed secondarily or surgically. Prior to initiation of NPWTi-d in this series, 46 of 131 (35%) patients had been receiving conventional NPWT, which was unsuccessful in promoting granulation tissue formation. Authors reported enhanced granulation tissue production with NPWTi-d using saline compared to conventional NPWT, in terms of filling the dead space more rapidly and completely. Undermined cavities and exposed bones were also more rapidly covered during NPWTi. The effects of instillation may have been more striking owing to systematic surgical debridement prior to initiating NPWTi-d and at each dressing change as appropriate.

Another study by Fluieraru and colleagues conducted a retrospective case review of 24 patients who were unsuccessfully treated with NPWT (n = 12) or who presented with complex wounds (n = 12) and then received NPWTi-d.^22^ Prior to NPWTi-d, all patients were surgically debrided. Saline was instilled for 30 seconds, followed by a 10-minute soak time and 4 hours of NPWT. Surgical closure was achieved in 23 of 24 wounds. Main observed clinical effects were promotion of granulation tissue formation and rapid filling of undermined cavities. Authors reported granulation tissue coverage changed from poor to quickly proliferating, filling the cavity and undermined areas during NPWTi-d in wounds that had previously failed conventional NPWT treatment. This work is supported by studies comparing granulation response of noninfected porcine excisional wounds that demonstrated thicker and higher quality granulation tissue after 6 to 7 days of NPWTi-d treatment with saline, versus NPWT alone,^23^^,^^24^ but these results have yet to be clearly confirmed in human studies.

Current authors recommend consideration of NPWTi in wounds with high levels of exudate and slough content, as well as acute traumatic wounds or acutely debrided wounds due to infected bone or soft tissue.^25^ In this series, saline was instilled for cleansing of most wounds (n = 39), with polyhexanide instilled in the remaining wounds that were more complex (n = 9). The addition of saline appeared to continually cleanse the wound base, add moisture to the wound, cleanse foam pores, and reduce pain at dressing changes. A recently convened consensus panel of expert users recommended use of NPWTi-d as adjunct therapy in acutely and chronically infected wounds, contaminated wounds, diabetic wounds, traumatic wounds, decubitus wounds, wounds with exposed bone, wounds with underlying osteomyelitis, painful wounds, and as a bridge between staged/delayed amputations.^26^ Instilled solutions most recommended with NPWTi-d by this expert consensus panel were polyhexanide and hypochlorite-based solutions.^26^ Other authors have reported successful use of NPWTi with saline, sterile water, and quarter strength's Dakin's solution.^22^^,^^27^^-^29

The economic benefits of NPWT have been demonstrated in studies that have applied cost dollars to clinical outcomes in randomized controlled trials^7^^,^^8^ or other comparative studies.^30^^,^^31^ Previous studies have also shown that early initiation of standard NPWT resulted in decreased costs for hospital stay^32^ and total and variable costs for traumatic patients^4^ as compared to late initiation of NPWT. It is interesting to hypothesize whether the patients receiving late NPWT would have benefited from NPWTi-d. Indeed, studies have shown that patients whose wounds failed to progress with standard NPWT showed improved healing with NPWTi-d.^21^^,^^22^ Our findings from the hypothetical economic model demonstrated a reduction in surgical debridement costs with NPWTi-d, owing to half of the trips to the OR as compared to NPWT. Although there were increased material costs with NPWT-d compared to NPWT, there were fewer therapy days with NPWTi-d, which translated into a significant cost savings.

The drastic differences in hospital stay, LOT, and time to closure observed between the 2 groups is influenced by a shift in protocol at our facility that gradually took place between 2010 when we treated the NPWT group and 2011–2012 when we treated the NPWTi-d group. Prior to incorporation of adjunctive NPWTi-d into our treatment regimen for infected wounds, these authors would initiate systemic antibiotics and NPWT, and then wait for a negative bacterial culture result and/or for granulation tissue coverage prior to surgical closure. The regular wound cleansing with NPWTi-d, in addition to debridement and systemic antibiotics, allowed us to rely more on sound clinical judgment in determining timing of wound closure, and we began closing wounds earlier, following the absence of additional purulence or necrotic tissue. In our series, duration of NPWTi-d was dependent upon the goals of the therapy, which included control of bacteria and/or wound bed preparation for closure. NPWTi-d was discontinued when the wound looked clean, there was no further gross contamination present, and the dressing had been in place for at least 48 hours since the last debridement. The method and timing of instillation of solutions may also play a role in effectiveness with NPWT, although comparative clinical evidence is clearly lacking in this area. We have observed excellent long-term closure results and reduction in hospital stay with this shift in practice and no longer consider negative cultures or granulation tissue coverage as required criteria for surgical wound closure.

This study has all of the limitations inherent in retrospective analysis, including potential selection bias, information bias, and missing data. Initial wound size data were not available for analysis and thus could not be considered in the demographic comparison. There are also likely unknown variables that could more accurately account for the differences in hospital stay and time to wound closure between the 2 groups.

## CONCLUSIONS

Our results from this retrospective study showed a reduction in hospital stay, mean time to wound closure, mean surgical debridements, and LOT using NPWTi-d. Furthermore, the reduced trips to the OR for surgical debridements and decrease in LOT translated into a cost savings with NPWTi-d compared to NPWT. We believe these findings warrant further study, and additional prospective, controlled studies are required to further assess the comparative cost-effectiveness of each treatment.

## Figures and Tables

**Table 1:** Patient demographics

	NPWTi-d (n = 48)	NPWT (n = 34)
Age, mean, y	42.7	41.5
Gender		
Female	24 (50%)	16 (47.1%)
Male	24 (50%)	18 (52.9%)
Wound type		
Upper extremity	13	11
Lower extremity	14	4
Trunk	21	19

**Abbreviations:** NPWT, negative pressure wound therapy; NPWTi-d, negative pressure wound therapy with instillation and dwell time.

**Table 2:** Comparative outcomes between NPWTi-d and NPWT patients

	NPWTi-d	NPWT	*P*
Patients, n	48	34	
Mean hospital stay, d	8.1	27.4	<0.0001
Mean time to wound closure, d	4.1	20.9	<0.0001
Mean surgical debridements in the OR	2	4.4	<0.0001
LOT, d	4.1	20.9	<0.0001

**Abbreviations:** d, days; LOT, length of therapy; NPWT, negative pressure wound therapy; NPWTi-d, negative pressure wound therapy with instillation and dwell time; OR, operating room.

**Table 3:** Potential cost-effectiveness of NPWTi-d

	NPWTi-d	NPWT	Difference
Patients, n	48	34	
Trips to OR for debridement	2.0	4.4	2.4
Mean cost of an OR debridement*	$3393	$3393	—
Total OR debridement cost	$6786	$14,929	$8143
Length of NPWTi-d, d	4.1	—	4.1
Length of NPWT, d	—	20.9	20.9
Daily cost of therapy†	$194.80	$106.08	$88.72
Total therapy costs	$799	$2217	$1418

*Granick et al.

†NPWTi-d system daily estimated cost—$194.80 (assumes daily cost of NPWT unit of $63.25, 0.8 canisters daily at $42.49 each, 3 medium dressings at $115 each/average hospital stay, and one cassette at $55 each; solution cost excluded. NPWT daily estimated cost—$106.08 (assumes daily cost of NPWT unit of $63.25, 3 medium dressings per week with mix of silver foam dressings (79%) at $71.96 each and black foam (21%) at $50.40 each, 0.3 canisters per day at $42.49 each).

**Abbreviations:** NPWT, negative pressure wound therapy; NPWTi-d, negative pressure wound therapy with instillation and dwell time; OR, operating room.
